# Metabolomic Studies of Lipid Storage Disorders, with Special Reference to Niemann-Pick Type C Disease: A Critical Review with Future Perspectives

**DOI:** 10.3390/ijms21072533

**Published:** 2020-04-05

**Authors:** Benita Claire Percival, Miles Gibson, Philippe B. Wilson, Frances M. Platt, Martin Grootveld

**Affiliations:** 1 Leicester School of Pharmacy, De Montfort University, The Gateway, Leicester LE1 9BH, UK; benita.c.percival@dmu.ac.uk (B.C.P.); miles.gibson@dmu.ac.uk (M.G.); 2 School of Animal, Rural and Environmental Sciences, Brackenhurst Campus, Nottingham Trent University, Southwell NG25 0QF, UK; philippe.wilson@ntu.ac.uk; 3 Department of Pharmacology, University of Oxford, Mansfield Rd, Oxford OX1 3QT, UK; frances.platt@pharm.ox.ac.uk

**Keywords:** metabolomics, lysosomal storage disorders, Niemann-Pick Type C Disease, lipidoses, nuclear magnetic resonance (NMR) analysis, liquid chromatography-mass spectrometric (LC-MS) analysis, multivariate power calculations, validation and cross-validation, biomarkers

## Abstract

Lysosomal storage disorders (LSDs) are predominantly very rare recessive autosomal neurodegenerative diseases.Sphingolipidoses, a sub-group of LSDs, result from defects in lysosomal enzymes involved in sphingolipid catabolism, and feature disrupted storage systems which trigger complex pathogenic cascades with other organelles collaterally affected. This process leads to cell dysfunction and death, particularly in the central nervous system. One valuable approach to gaining insights into the global impact of lysosomal dysfunction is through metabolomics, which represents a discovery tool for investigating disease-induced modifications in the patterns of large numbers of simultaneously-analysed metabolites, which also features the identification of biomarkers Here, the scope and applications of metabolomics strategies to the investigation of sphingolipidoses is explored in order to facilitate our understanding of the biomolecular basis of these conditions. This review therefore surveys the benefits of applying ’state-of-the-art’ metabolomics strategies, both univariate and multivariate, to sphingolipidoses, particularly Niemann-Pick type C disease. Relevant limitations of these techniques are also discussed, along with the latest advances and developments. We conclude that metabolomics strategies are highly valuable, distinctive bioanalytical techniques for probing LSDs, most especially for the detection and validation of potential biomarkers. They also show much promise for monitoring disease progression and the evaluation of therapeutic strategies and targets.

## 1. Introduction

**Figure 1:**
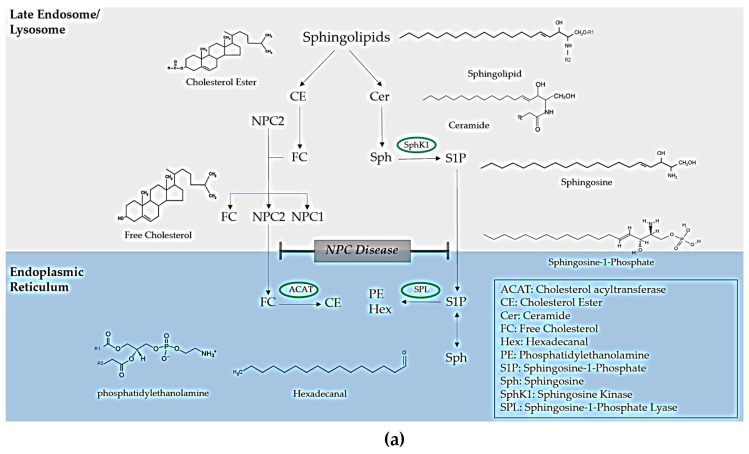

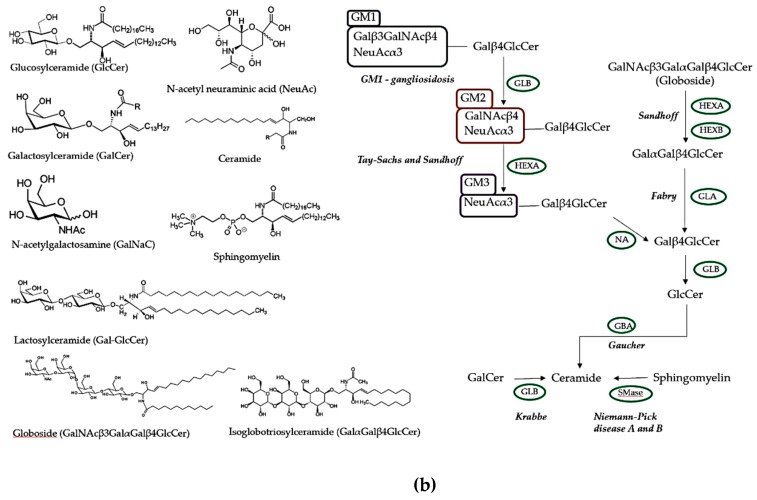
Metabolism of lipidoses (**a**) Impact of lipid trafficking in Niemann-Pick type C disease. (**b**) Impact of lipid metabolism in other lysosomal storage disorders. Abbreviations: ACAT, Cholesterol acyltransferase; CE, Cholesterol Ester; FC, Free Cholesterol; Hex, Hexadecanal; PE, Phosphatidylethanolamine; S1P, Sphingosine-1-Phosphate; Sph, Sphingosine; SphK1, Sphingosine Kinase; Sphingosine-1-Phosphate-Lyase; GlcCer, Glucosylceramide; NeuAc; *N*-acetyl nueraminc acid, GalCer, Galactosylceramide; Gal-GlcCer, Lactosylceramide; GalNAcβ3GalαGalβ4GlcCer, Globoside; GalαGalβ4GlcCer, Isoglobotriosylceramide.

In the functional lysosome, lipids and other macromolecules, such as carbohydrates, peptides, and nucleic acids, are degraded through the action of acid hydrolases. Inherited defects in genes that encode lysosomal enzymes and non-enzymatic proteins can cause the build-up macromolecules within the late endocytic system [1]. The ‘umbrella’ term for these diseases is lysosomal storage disorders (LSDs), a classification that can be further sub-categorised into lipid storage disorders (lipidoses), mucopolysaccharidoses, glycoprotein storage disorders and mucolipidoses. The lipidoses are the focus of this review, and are listed in Table 1, together with their corresponding defective genes encoding the specific enzyme/protein which is depleted or malfunctional. Table 1 provides detailed information regarding treatment options, including enzyme replacement therapy (ERT), which is used to remove the accumulation of the respective lipids by enzymatic processes; substrate reduction therapy (SRT), which is employed to remove the accumulation of the respective lipids by the use of a drug or binding molecule; chaperone therapy, which aims to ‘scaffold’ the protein so it is no longer unfolded and functions appropriately; and finally, haematopoietic stem cell therapy, which involves the implantation of stem cells. Niemann-Pick disease type C (NPC) is caused by mutations in two genes *NPC1* (95% of clinical cases) and *NPC2* and exhibits storage of multiple sphingolipids and cholesterol. The lysosomal storage of sphingosine in NPC gives rise to a loss of lysosomal calcium ions, a process accompanied by the accumulation of unesterified cholesterol and glycosphingolipids [2,3]. *NPC1* encodes a large trans membrane protein that resides in the limiting membrane of the lysosome and is termed NPC1 protein [2]. *NPC2* encodes a small soluble lysosomal protein that binds cholesterol within the lysosomal lumen, and which is believed to transfer cholesterol to the NPC1 protein. Full details concerning how such proteins may complement each other to permit cholesterol passage through the glycocalyx and the lysosomal membrane are available in [2]. Figure 1a shows how NPC disease impacts on the trafficking of lipids, whereas Figure 1b shows mechanistic schemes depicting the pathogenesis of a series of other lipidoses which arise from of enzymatic defects, unlike trafficking disorders as in NPC disease.

The applications of multivariate (MV) metabolomics strategies have been comprehensively reviewed for many diseases [4], for example cancer [5,6] and Parkinson’s Disease [7]. Links between LSDs and more common neurological disorders have also been described [1,3].

Metabolomics represents the investigation of a wide range of metabolites simultaneously within selected biological system. A plethora of such systems can be explored, and which may be microscopic, such as a living cell, or alternatively, tissues and organs biopsies, and biofluids, e.g., blood plasma and urine. A metabolomics strategy is advantageous, since the chosen system can be analysed at the point of response after exposure to specific stimuli, for example treatment or exercise regimens, under pre-set conditions determined by the experimental design. Metabolomics assists with allowing researchers to develop a deep understanding of how the system under study responds to such stimuli and in clinical studies, this has potential applications for the monitoring of patient diagnosis and prognosis, therapeutic management and even drug development. Metabolomics is typically performed using highly reproducible and specialised multicomponent analytical techniques. 

Our major focus in the present review will be placed on high-resolution nuclear magnetic resonance (NMR) spectroscopic, and mass-spectrometric (MS) including liquid chromatographic-mass spectrometric (LC-MS) and LC-MS-MS tandem analyses. Applications of metabolomics techniques to LSD biomarker discovery have markedly increased recently, and these are summarised in Table 2. 

The multicomponent analytical ability of high-resolution NMR analysis technologies offers major advantages which are not often readily achievable by alternative techniques such as LC-MS, which although of greater sensitivity, are focused on the targeted measurement of only one or several chromatographically related biomarkers. Indeed, the application of a series of n (for example, ≥ 6) biomarker concentration variables in a metabolomics context offers major advantages over the detection and determination of only a single biomarker variable. First, biofluid or tissue biopsy ‘patterns’ or ‘signatures’ of metabolites which are representative of a particular LSD process (and potentially encompassing a range of such biomarkers, each with highly significantly up- or downregulated concentrations) will, at least in principle, provide a much higher level of confidence regarding the diagnosis, prognosis and response to treatment of such disorders than that realisable from a single biomarker; second, the metabolic patterns detected and determined, together with their correlations to particular components or factors [linear and/or sometimes quadratic combinations of predictor biomarker variables] provide extensive and valuable information regarding the nature of the metabolic stress response(s) or disturbance(s), e.g., pathogenic imbalances in amino acid, amino-sugar, fatty acid and nucleotide metabolism, TCA cycle intermediates, etc., and also the class of organ and tissue damage (e.g., neurodegeneration), together with the sub-cellular localizations involved; third, the identification of ‘suppressor’ variables, i.e., those which do not directly provide disease classification information but nevertheless exert a significant and sometimes substantial influence on reliable biomarker level variables themselves, are also detectable using correlated component regression (CCR) approaches [8]. 

This review will critically consider the recent history and applications of metabolomics analysis strategies to the biomolecular investigation of sphingolipidoses. For the purpose of this review, NPC1 will be considered as a sphingolipidosis, because in addition to cholesterol it accumulates sphingolipids, and since it shares symptoms and pathogenic mechanisms with the sphingolipidoses Niemann-Pick types A and B.

## 2. Metabolomic Evaluations of Sphingolipidoses

### 2.1. Niemann-Pick Diseases 

The majority of metabolomics research conducted on sphingolipid storage diseases has been focused on NPC1 disease. High-resolution ^1^H NMR analysis has been successful in identifying potential urinary biomarkers for NPC1 disease diagnosis, and these include branched-chain amino acids (BCAAs), selected bile acids and 3-aminoisobutyrate [22], the latter arising from either BCAA catabolism or thymine degradation. These analyses further indicated that the brain and liver were the prominent sites affected, an observation which is consistent with its disease manifestations, including seizures and hepatomegaly. The area under the receiver-operating characteristic curve (AUROC) values determined ranged from 0.81–0.91 for the most significant metabolites, and partial least squares-discriminatory analysis (PLS-DA) yielded significant values of Q^2^ (0.56) and an accuracy of 0.93 [22]. ROC curves are plots of the true positive classification rate (i.e., sensitivity or probability of detection) against the false positive rate (1-specificity). AUROC values can range between 0 and 1.0, 1.0 representing perfect classification test success, with 0.5 no success whatsoever; values ranging within the 0.5 – 1.0 range indicate increasing levels of classification success on consideration of sensitivity and specificity (however, for all AUROC values determined, it is necessary to ensure that their lower 95% confidence interval (CI) values do not cover the null 0.5 value).Q^2^ represents the R^2^ value (the latter quantitatively reflecting extent to which independent multivariate components explain the dependent one, qualitative or quantitative) when a PLS model constructed from a training set is applied to an unknown ‘test’ set. Hence, a high value for Q^2^ is one with minimal deviation from R^2^, and this indicates that the PLS model developed functions independently of the dataset employed to train it. 

The above NMR-based metabolomics approach also has potential applications for disease progression monitoring, but these benefits have yet to be demonstrated in practice. At the sub-cellular level, the lysosome, ER and mitochondria were the main affected organelles in this investigation. Moreover, high-performance liquid chromatographic (HPLC) analysis coupled with a UV detection system has also been used to target specific amino acid imbalances and DNA methylation in murine cerebellum collected from experimental mice with this condition, in which BCAAs significantly (*p* > 0.05) arose from elevated glutamine/glutamate oxidation, in a response to prolonged neuron survival [53]. The combination of DNA methylation and amino acid imbalances explores new mechanisms which are ascribable to neurodegeneration in NPC disease. Indeed, this combinatorial approach could influence new treatment strategies, in which these amino acid imbalances are addressed by specific treatments. 

LC-MS analysis has been used to understand upregulations or downregulations of potential biomarkers, as well as changes in biomarker expression in NPC1 disease as a response to treatment with miglustat and 2-hydroxypropyl-β-Cyclodextrin (HPβCD) [9]. The mechanism of action for miglustat is its inhibition of glucosylceramidase (GlcCer) synthase activity, whereas the mechanism of action for HPβCD features reductions in cholesterol and overall lipid accumulation through the formation and transport of inclusion complexes; the hydrophobic interior of HPβCD incorporates hydrophobic biomolecules such as cholesterol to form such inclusion complexes. This study monitored the effectiveness of drug administration and uptake, and identified specific upregulated markers in mouse brain, liver and spleen. These included elevated levels of ceramide, GM1, GM3, sphingolipid species, and lactosylceramide in NPC1 tissues, which markedly reduced upon treatment with HPβCD, an effect greater than that observed with miglustat treatment [9]. Elevated markers in human plasma and cerebrospinal fluid (CSF) were identified as monohexosylceramides and ceramides [9]. Administration of the substrate reduction therapy (SRT) miglustat which inhibits GlcCer synthase did not significantly reduce these biomarker concentrations. [9]. However, it was proposed they may downregulate on administration of HPβCD, since this downregulation was observed in the tissues of the mouse model [9]. This research needs further confirmation of biomarkers prior to translation into clinical practice for disease diagnosis or clinical progression. Another research study monitored the effectiveness of HPβCD treatment in mice, using 2,4(S)-hydroxycholesterol (2,4(S)-HC) as a pharmacodynamic marker, employing an LC-MS-MS approach to assess the metabolic activity in NPC1 disease [54]. Brain 2,4(S)-HC levels were found to build-up from high cholesterol levels. 2,4(S)-HC is produced exclusively at this location, and acts as a biological response to eliminate cholesterol; excess cholesterol is toxic, and 2,4(S)-HC can cross the blood-brain barrier (BBB), unlike cholesterol itself [54]. A different investigation, [55] also monitored 2,4(S)-HC levels in blood plasma in order to assess treatment outcomes with HPβCD in murine models; 2,4(S)-HC was therefore proposed as a biomarker for diagnostic purposes and therapeutics development. 

Oxidation products of cholesterol are commonly monitored biomarkers in NPC disease, not only because of the increased levels of intercellular free cholesterol, but also in view of oxidative stress which is a feature of the condition. Similarly, selected bile acids also serve as disease biomarkers; since these are also synthesised from cholesterol in the liver, they have been correlated with cholestasis in NPC1 patients [15,22]. One study, [15], has reported increases of cholestane-3β5α6β-triol levels in NPC1 patient plasma profiles, and in mouse plasma and liver, whole brain and cerebellum tissues. Cholestane-3β5α6β-triol, a further oxidation product of cholesterol, which functions as an endogenous neuroprotectant, has been reported to be upregulated in the blood plasma of NPC patients [10,12,21,28]. Other disorders, such as lysosomal acid lipase deficiency and cerebrotendinous xanthomatosis, have also been shown to correlate with enhanced cholestane-3β5α6β-triol concentrations in human plasma, and hence this biomarker suffers specificity issues for the diagnosis of NPC1 disease [28]. Others indicated that cholestane-3β5α6β-triol could be used as a biomarker in conjunction with lyso-sphingomyelin-509, an approach which provided a model with 91% specificity and 100% sensitivity [21]. These findings were validated using HPβCD, with cholestane-3β5α6β-triol significantly downregulated with treatment [15]. However, such critical effective drug-based validation processes were not performed in the studies of [10,12,21,28].

Two investigations, [10,15], also reported an elevation in 7-ketocholesterol, which is also an oxidation product of cholesterol, indicating that 7-ketocholesterol may be non-specific as a biomarker for diagnosis, as suggested in other studies [26,28]. Human plasma 7-ketocholesterol concentrations were also found to be elevated in NPB, sterol disorders, peroxisomal diseases, metachromatic leukodystrophy, mucolipidosis II/III, LAL-deficiencies [28] and cardiovascular diseases [56]. Notably, other correlations between NPC and NPB patients have been noted, since both exhibit elevated levels of lysosphingomyelin in blood plasma and dried blood spots as a diagnostic marker [21,22] respectively. Indeed, current literature predominately focuses on biomarkers known to be associated directly with the disorder, and the accumulation of intracellular metabolites, rather than what effect this accumulation has on the metabolome as a whole. A combination of statistically significant biomarkers, such as cholesterol oxidation products, bile acids and 3-aminoisobutyrate, including those which are directly and indirectly associated with the disease, could offer a more sensitive and specific solution for the long-term monitoring of LSDs [17]. Other potential sphingomyelin precursors have been investigated including sphingosylphosphorylcholine, which has been demonstrated to be marginally increased in NPC plasma using an LC-MS-MS strategy in a pilot study [11]. However, it was recommended that this biomarker should be used in conjunction with, for example, plasma lysosphingomyelin-509, also known as *N*-palmitoyl-O-phosphocholineserine [57] (with a median fold-change value of 65 in NPC plasma relative to healthy controls), in order to be applied as a biomarker for diagnostic purposes in clinical practice [11]. 

Further studies have been performed using LC-MS-based methodologies, analysing no more than two NPC samples which monitored urinary bile acid sulphate conjugates for diagnostics, i.e., 3β-sulfooxy-7β-hydroxy-5-cholen-24-oic acid and 3β-sulfooxy-7-oxo-5-cholen-24-oic acid, both of which were found to be significantly upregulated in NPC disease [14,18,57]. However, more recently, a larger study performed by the same research group demonstrated upregulations in 3β-sulfoxy-7β-hydroxy-5-cholen-24-oic acid and its glycine and taurine conjugates, using urine samples from NPC patients (*n* = 23) and control (*n* = 28) participants [19]. A further investigation with control (*n* = 38) and NPC patients (*n* = 28) found similar findings with an AUROC value > 0.92 for 3β-sulfoxy-7β-hydroxy-5-cholen-24-oic acid, 3β-sulfoxy-7-oxo-5-cholen-24-oic acid, non-amidated 3β-sulfooxy-7β-*N*-acetylglucosaminyl-5-cholen-24-oic acid, glycine-amidated 3β-sulfoxy-7β-*N*-acetylglucosaminyl-5-cholen-24-oic acid, and taurine-amidated 3β-sulfoxy-7β-*N*-acetylglucosaminyl-5-cholen-24-oic acid [20]. The most significant marker with an AUROC value of 1.0 was 3β-sulfooxy-7β-hydroxy-5-cholen-24-oic acid [20]. The sensitivity and specificity of these biomarkers, and how treatments can use the biomarkers to provide diagnostic information, needs to be explored. 

Bile acids have been identified as potential biomarkers for early diagnosis in view of the observed upregulation of their precursors 7-oxocholesterol and cholestane-3β5α6β-triol in human urine and plasma [27]. A novel MS assay was created in order to detect these bile acid biomarkers in dried blood spots, urine and plasma, and with a much larger sample size than those featured for investigation of the above dysfunction [27]. However, conclusive evidence demonstrating a decrease in these biomarker concentrations following treatment is still lacking [27]. 

A combination of these biomarkers in selected biofluids could provide more statistical power, since the majority of the analyses performed in these studies were targeted and univariate, while MV approaches could provide improved specificity for NP disease diagnostics. However, few investigations have correlated these markers with disease progression and disease severity indices. Clearly, correlations of such biomarker levels with disease profiles is necessary for successful clinical management [58]. It is, therefore, necessary to ensure that potential biomarkers selected for NPC1 disease are specific to this condition in order to avoid misdiagnoses. At present, there is a lack of evidence in the literature showing the intrinsic value of these candidate biomarkers individually. Indeed, most of these diseases exhibit subtly different manifestations, translating into modified metabolic responses. Therefore, without thorough testing of the range of other LSDs, and without the rigorous validation of such biomarkers, misdiagnosis remains a possibility.

Studies performed on Chinese hamster ovary cells, and cells collected from an NPC patient versus an NPC carrier, and also COS-7 and fibroblast cells from an NPC patient, have also been analysed using an HPLC electrospray ionisation (ESI)/MS/MS strategy to determine sphingomyelin levels [59]. This study demonstrated an upregulation in total sphingomyelin, including specific species, such as d18:1/18:1, d18:1/18:0, d18:1/20:0, d18:1/22:0, d18:1/24:1, and d18:1/26:1 [59]. Moreover, this investigation showed that Rab9, a GTP-binding protein that controls trafficking from the late endosome to the Golgi, is defective; upon reducing sphingomyelin within the cell using sphingomyelinase, Rab9 was able to function more effectively, and cholesterol accumulation was concomitantly diminished significantly [59]. Consequently, sphingomyelin reduction is another potential therapeutic target for NPC disease. This investigation demonstrated the usefulness of metabolomics analysis of cell systems in lysosomal storage disorders to assess the mechanisms of cellular pathogenesis. 

Finally, high-resolution NMR analysis has been employed to observe the urinary excretion of miglustat, valproate, and valproate metabolites in NPC1 disease [60]. Valproate is frequently used in NPC1 disease to control seizures. Hence, this NMR-based approach could contribute to more personalised medicine approaches in human patients. Similar studies have also been performed with HPLC coupled with light scattering as a detection system (HPLC-LS), and these demonstrated the detection of HPβCD in human urine [61].

### 2.2. Fabry Disease

Fabry Disease (FD) (*OMIM 301500*) is an X-linked disorder arising from a defect in *GLA*, causing dysfunction with α-galactosidase A and the storage of Gb3 [1,62]. Targeted approaches have been applied for the potential diagnosis of FD using MS methodologies and have been successful using a relatively large sample size (Table 2) [29,30,33]. Typical diagnosis for Fabry disease involves an enzymatic assay of α-galactosidase A activity in leukocytes using a blood sample, or detection of Gb3 deposition in a skin biopsy, followed by a genetic test. Recently, in a cohort of Chinese patients the detection and determination of globotriaosylsphingosine (lyso-Gb3) in blood plasma provided a more significant correlation with the Mainz severity score index than α-galactosidase A activity measurements did, i.e., r = 0.71 and -0.69 for globotriaosylsphingosine and α-galactosidase A, respectively [32]. In particular, this study established that lyo-Gb3, analysed using LC-MS-MS, was more successful in diagnosing female FD participants [32]. Diagnosis of females is a particularly challenging issue in view of the X-linked inheritance pattern of this disorder. Females demonstrated a later onset of FD, with the difference in onset in males and females being 13 and 33 age yrs. respectively [32]. These previous investigations, along with reports from [36], which examined the plasma of over 250 FD participants (some of which received enzyme replacement treatment (ERT)), suggested a causal link between increased levels of lyso-Gb_3_ and FD. However, this biomarker cannot be applied to young patients since age has a significant impact on the concentration of lyso-Gb_3_ monitored [36].

Moreover, the levels of plasma lyso-Gb3 and four other analogues (with m/z values of 786, 784 (−2), 802 (+16) and 804 (+18)) have been recently used to assess treatment outcomes using LC-MS-MS in a mouse model, in addition to six isoforms of Gb3 including C16:0, C16:1, C18:0, C22:0, C24:1, C24:0 [41]. The novel human α-galactosidase A mRNA treatment using human α-galactosidase A showed dose-dependent activity in lyso-Gb3 in mice, and lyso-Gb3 concentrations were significantly reduced after a 6-week period [41]. However, this investigation only used male mice aged 5 months to assess this treatment. In another recent study, it was found that Gb3 and isoforms were unable to distinguish between female and male FD participants versus controls, but good discrimination was established between male control and male FD participants [63].

A non-invasive method using urine sampling has been explored using LC-MS [33,34,35]. A FD-specific biomarker, lyso-Gb3, is significantly elevated in FD patients relative to that in healthy controls [33,34,35]. This biomarker is also sensitive to the gender of the patient; indeed, female patients were less likely to be diagnosed with this X-linked LSD in view of lower observed levels, and therefore results arising from female samples are susceptible to misdiagnosis. Despite this, lyso-Gb3 has been suggested to serve as a sensitive biomarker in urinary profiles; however, the patient cohort involved was not paediatric, and this marker has not been demonstrated to be an early marker for FD [31]. Notwithstanding, there are some issues associated with using biofluids from single gender donor groups, data acquired not being fully representative of the entire sample population [33]. However, since FD is X-linked and more severe in male patients, this approach is considered appropriate for pilot studies in FD. Furthermore, age-matched samples also offer substantial diagnostic potential, since some markers, such as lyso-Gb_3,_ are undetected in younger patients, as shown by [34]. This study also reported the misdiagnosis of male and female participants through the use of a single biomarker, where ideally a universal methodology or the use of reference ranges would be the preferred approach [34]. Using a range of control, diseased, and heterozygous participants improves the validation of biomarkers [17], and global standards in the field have been proposed in order to ensure that data acquired from such studies are clinically reliable [64]. 

More recently, it has been demonstrated that there are potential urinary and plasma markers in the more challenging FD female cohort using LC-MS-MS [42]. Ceramide dihexosides were proposed as biomarkers, including C22:1, C22:0, C22:1-OH, C22:0-OH, C24:2, C24:0, C24:2-OH, C24:1-OH, C24:0-OH, C26:0 Gb3 isoforms, and these were observed to be 5-fold elevated in the urinary profiles of asymptomatic FD patients [42]. However, this significance was not observed in plasma profiles [42]. This study was the first to implement principal component analysis (PCA) and orthogonal partial least squares-discriminatory analysis (OPLS-DA) MV analysis strategies in order to identify potential biomarkers in FD, in addition to standard univariate comparisons. FD urine samples have also been analysed using electrospray ionization-mass spectrometry (ESI-MS) in [65], and upregulations in ceramide trihexoside, lactosylceramide and ceramide, along with downregulations in glucosylceramide and sphingomyelin, were observed. These markers were suggested to be ideal for the detection of FD prognosis for patients receiving enzyme therapy [65].

Validation must be performed prior to translation into clinical practice, or alternatively the rigorous establishment of an age and/or gender-specific reference range of biomarker concentrations is required. Furthermore, it would be advantageous to establish a global metabolomics approach for urine and plasma samples using ^1^H NMR or alternative multicomponent analysis in order to ascertain the validity of the results and/or the uniformity of similar biomarkers potentially observable across the entire metabolome. Biomarkers can be validated through pharmacokinetic models, which show that they normalise, or start to normalise, following treatment. Moreover, specificity and sensitivity of the biomarker to a specific lipidoses, along with an overall evaluation of the reliability of analytical equipment and methods employed, notably that concerning the accuracy, precision, reproducibility and limits of biomarker detection and quantification, are further important parameters for consideration.

### 2.3. Gaucher Disease

Similar to NPC disease, there are variants of Gaucher disease (GD) (types 1, 2 and 3): GD 1 (*OMIM 230800*) is non-neurological, while GD 2 (*OMIM 230900*) and GD 3 (*OMIM 231000*) are neurological conditions also involving the spleen and liver [66,67]. Targeted metabolomics approaches have also been applied to GD, using LC and LC-MS technologies. The plasma biomarker glucosylceramide (GlcCer), and ceramide ratios, have been used in combination with ERT treatment for this condition, with levels significantly decreasing with corresponding treatments in GD 1 patients [44]. Moreover, glucosylsphingosine has also been determined to be a biomarker in dried blood spots (DBS) from children with GD 1 and 3 diseases [47]. Glucosylsphingosine has also proven to have diagnostic potential in other LSDs, further validating its potential as a generalised LSD-specific biomarker [45,46]. However, glucosylsphingosine levels are also markedly elevated in the plasma of patients with NPC disease, but to a different extent—GD concentrations of this plasma biomarker are relatively higher, as noted in Ref. [24]. Another investigation showed an upregulation of glucosylsphingosine in KD and GD plasma samples, but the concentrations were distinguishable, since GD patients had significantly higher levels [68]. As with any potential screening biomarker, the selection of glucosylsphingosine would be required to involve the provision of essential reference ranges in order to ensure that the correct diagnosis decision was made for both GD and NPC patients. Urine has received little attention as a potential biofluid sample type for GD metabolomics studies, and this is reflected by the lack of reports available in the area. 

Another investigation reported a potential marker for GD in serum, specifically GlcCer. Notwithstanding, there were validation issues in view of the lack of controls and heterozygous carrier samples integrated into the study for comparative purposes [43]. Indeed, statistical information from a sample size of only *n* = 2 is, of course, unreliable [43]. However, such small sample sizes represent one of the challenges presented by the monitoring a rare LSD in human participants. Furthermore, samples analysed in this study were collected using invasive methodologies, such as venipuncture. Invasive sample collection is a common theme in the observation and monitoring of LSDs. However, more evidential data could be generated by considering urinary metabolic profiles, together with those of other readily accessible biofluids such as saliva, perhaps, which have successfully been used for the diagnosis of other diseases, including cancer and diabetes [69]. 

### 2.4. Krabbe Disease

To date, few human metabolomics investigations in Krabbe Disease *(OMIM 245200)* (KD) have been performed using multicomponent LC-MS or NMR analyses [70]. Such prospective metabolomics studies may require a period of years to perform, e.g., the DBS analysis of samples collected over a 6-year period from patients with KD [49], and a similar dried blood spot (DBS) analysis collecting samples over a 5-year period from KD patients [71]. One study completed targeted analysis on pyschosine [45], a biomarker indicator of impaired galactosylceramidase, and have provided recommendations and reference ranges for KD patients. Indeed, others observed changes in the metabolism of twitcher KD mice hindbrain tissue, specifically decreased phospholipid turnover, mitochondrial metabolism of BCAAs, and upregulated neurotransmitter metabolism, inflammatory cascades and osmolytes, when compared to those of healthy controls [48]. Another investigation has assessed pyschosine levels in Krabbe’s patients and newborns against ‘at risk’ new-borns [50]. Additional investigations are required, particularly against alternative control samples, along with larger sampling sizes to validate these findings. An additional investigation compared KD patients to age-matched healthy controls and found a significant elevation of pyschosine in DBS collected from KD patients, although there was also an increase in carriers [51]. At present, there are no treatment strategies available for KD patients, which presents a major barrier against successful biomarker validation.

### 2.5. GM1, GM2 and GM3 Gangliosidoses 

Although much is known regarding the clinical aspects of the GM1 *(OMIM 230500)* (GM1), and GM2 gangliosidoses (*OMIM 268800*) (GM2), the cellular mechanisms leading to neurodegeneration are poorly understood, and the precise functions of gangliosides (i.e., their cellular and biochemical properties) are not yet fully understood [72,73]. Moreover, very few metabolomics studies focused on the GM1 and GM2 gangliosidoses have been performed.

More recently, reverse-phase LC has been used to analyse biomarkers in biofluids; these can also be measured in brain and liver tissues collected from mice, and hippocampus samples from humans with Sandhoff (SD, GM2) disease [74]. PCA analysis was able to successfully distinguish between SD and control hippocampus samples from humans, in addition to brain and liver samples from control and SD mice [74]. Disturbances were observed in pathways associated with protein catabolism and lipid metabolism. *N*-acetyl compounds were altered in the SD brain from humans and mice, including elevated *N*-acetylgalactosamine 4-sulphate which has been proposed to be correlated with a deficiency in the HexB enzyme [74]. *N*-Acetyl-l-aspartate and l-glutamate were downregulated in the brain [74]. The investigation focused on brain samples and provided potential new targets for biomarkers which may be accessible in non-invasively collected biofluids. 

A study distinguished between different gangliosides; however, it unfortunately did not account for the matrix effects exerted by biofluids since the experiments were performed in vitro [75]. Another investigation went further and developed a liquid chromatography-electrospray ionisation-mass spectrometry (LC-ESI-MS) method which was able to monitor blood serum ganglioside levels in participants (*n* = 10) with different oesophageal diseases, but not the gangliosidoses [76]. Other authors, [77], have developed a method for the observation of GM1 and GM2 gangliosides simultaneously in CSF using LC-MS-MS. This methodology used 30 human CSF samples in which GM1 and GM2 were ‘spiked’ and recovered with a high accuracy, i.e., 98 and 102 % respectively. More recently, quantitative immunoassays were used to determine key biomarkers in CSF and blood serum in 2 infantile Sandhoff, 9 infantile Tay-Sach’s, 5 juvenile Sandhoff, 6 infantile GM1, 7 late infantile GM1 and 1 juvenile GM1 patients, and compared these to 100 healthy control participants [78]. Of 188 metabolites tested, five key markers identified were epithelial-derived neutrophil-activating protein 78, monocyte chemotactic protein 1, macrophage inflammatory protein-1 alpha, macrophage inflammatory protein-1β, and tumour necrosis factor receptor 2, which were all upregulated in the CSF of gangliosidoses infantiles, and were also linked to inflammation within the central nervous system (CNS). In view of their rarity and severity, longitudinal metabolomics studies will, of course, take longer to perform. Markers which could be evaluated include those pertaining to CNS degradation, or lysosomal dysfunction such as quinolinate and *N*-acetylsugars respectively. 

Another investigation explored urinary *N*-acetylated storage compound biomarkers in patients with GM1 disease which arises from β-D-galactosidase deficiency [52]. In this study, TLC separations were sequentially followed by 1D ^1^H NMR analysis [52]. The first of two bands investigated in this manner corresponded to an *N*-acetylated biantennary octasaccharide, and the second to an *N*-acetylated triantennary decasaccharide or tetraantennary dodecasaccharide [52]. The 500 MHz ^1^H NMR profile of a patient with this condition contained a series of *N*-acetyl-CH_3_ function singlet resonances within the 2.02–2.04 ppm range, the band of highest intensity corresponding to δ = 2.03 ppm [52]. These results are consistent with elevations in the urinary concentrations of oligosaccharides with a D-galactosyl residue at their non-reducing ends. 

^1^H NMR spectra acquired on urine collected from a patient with Sandhoff disease demonstrated the excretion of oligosaccharides, specifically acetamido-CH_3_ function singlet resonances within the δ = 2.02–2.08 ppm range, the highest intensity one corresponding to a concentration of ca. 80 µmol/mmol of creatinine [52]. Combined preparative TLC-^1^H NMR analysis provided evidence that these signals were at least partially ascribable to oligosaccharides with the structures β-GlcNAc-(1→2)-α-Man-(1–3)-[β-GlcNAc-(1→2)-α-Man-(1–6)]-β-Man-(1→4)-GlcNAc (band 1), and β-GlcNAc-(1→2)-α-Man-(1–3)-[β-GlcNAc-(1→4)][β-GlcNAc-(1→2)]-α-Man-(1–6)]-β-Man-(1→4)-GlcNAc (band 2) [52]. 

**Figure 2:**
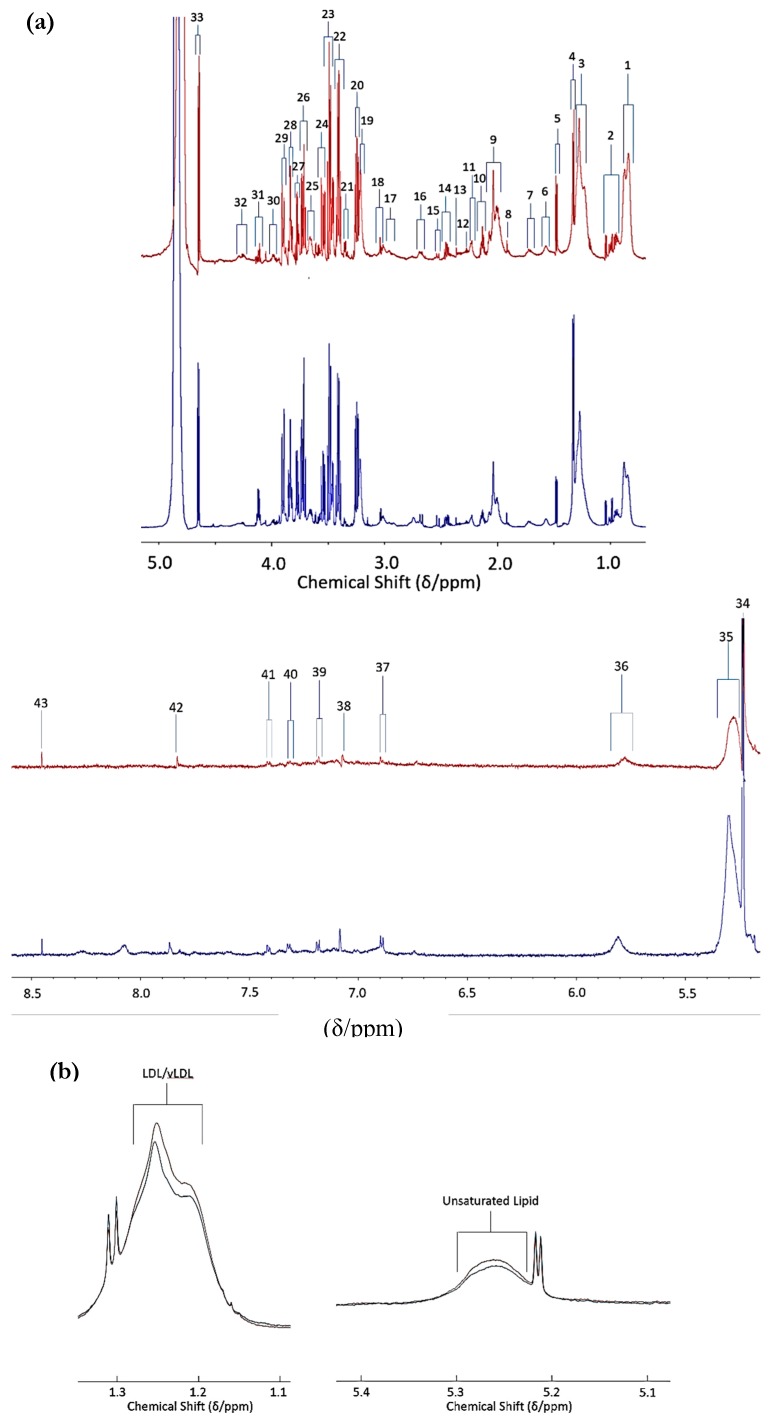
GM1 Type II and control plasma NMR profiles. (**a**) Typical 700 MHz ^1^H NMR spectral profiles from GM1 Type II gangliosidosis and corresponding control plasma (blue and red spectra respectively). Assignments: [1] very-low-density-lipoprotein (vLDL)/low-density-lipoprotein (LDL) triacylglycerol (TAG)-terminal-CH_3_ functions; [2] BCAAs (valine-, leucine- and isoleucine-CH_3_s); [3] vLDL/LDL-bulk-chain-(-CH_2_-)_n_; [4] Lactate-CH_3_; [5] Alanine-CH_3_; [6] lipoprotein TAG-CH_2_CH_2_CO- [7] unassigned multiplet; [8] Acetate-CH_3_ [22] acute-phase glycoprotein-carbohydrate side-chain *N*-aceytylsugar-CH_3_/lipoprotein TAG-CH_2_-CH=CH-; [53] glutamine-β-CH_2_; [9] lipoprotein TAG-CH_2_-CO_2_-; [54] acetone-CH_3_; [55] acetoacetate-CH_3_; [15] glutamine-γ-CH_2_; [10] citrate-CH_2A/B_; [12] lipoprotein TAG-CH=CH-CH_2_-CH=CH-/citrate-CH_2A/B_; [28] albumin lysine residue-ε-CH_2_; [21] free lysine-ε-CH_2_/creatinine-N-CH_3_/creatine-N-CH_3_; [26] high-density-lipoprotein phospholipid choline head-group-N^+^(CH)_3_; [56] β-Glucose-C2H; [24] unassigned multiplet; [11,14,17,18,25] glucose-C2–6H ring protons; [57] unassigned multiplet; [20] tyrosine-/histidine-/phenylalanine-α-CH’s; [27] lactate-CH; [58] threonine-α-CH; [59] β-glucose-C1H; [60] α-glucose-C1H; [61] lipoprotein TAG-CH=CH-; [62] urea-CO-NH_2_; [29,30] tyrosine aromatic-CH protons; [33,41] histidine-imidazole ring-CHs; [32,36] phenylalanine aromatic-CH’s; [41] Histidine-CH [63] formate-CH. (**b**) Average blood plasma ^1^H NMR profiles of unfasted GM1 Type II gangliosidosis patients (*n* = 10) (blue) and fasted control participants (*n* = 27) (red). Left: elevated vLDL/LDL TAG- bulk-chain-(-CH_2_-)_n_ resonance intensities in healthy controls (δ = 1.10–1.30 ppm)/ Right: elevated unsaturated TAG-CH=CH- resonance intensities in healthy controls (δ = 5.10–5.40 ppm). The Supplementary Materials section provides details regarding ethical approval and informed consent of participants, ^1^H NMR sample preparation and acquisition parameters.

Recently, our group performed a pilot investigation using a conventional ^1^H NMR-based metabolomics approach. This previously unpublished pilot study was conducted in order to identify new, potentially valuable biomarkers for GM1 Type II gangliosidosis, particularly those which correlated with severity and progression. Plasma samples were collected from patients with GM1 Type II Gangliosidosis (*n* = 10) and control participants (*n* = 28) with mean ages of 11 ± 2.3 and 22 ± 0.7 years, respectively (Figure 1). More than 40 metabolites were detectable in these spectra, including a range of biomolecules such as short-chain organic acid anions (e.g., acetate, formate, fumarate, lactate, pyruvate, succinate, glycolate and 3-D-hydroxybutrate); the BCAAs leucine, isoleucine and valine; other amino acids including glycine, alanine, glutamate, glutamine, lysine, proline, taurine, histidine, phenylalanine and tyrosine, etc., together with *N*-acetylamino acids; carbohydrates, most especially GM1 disease-relevant *N*-acetylsugars; and nicotinate and nicotinamide pathway metabolites, among others. Typical spectra acquired are shown in Figure 2a. Average spectral intensity profiles, which show significant differences in lipoprotein-associated triacylglycerol concentrations between the two phenotypes, are depicted in Figure 2b. ^1^H NMR spectra therefore clearly reveal disturbances in lipid metabolism, a common feature of LSDs. 

**Figure 3:**
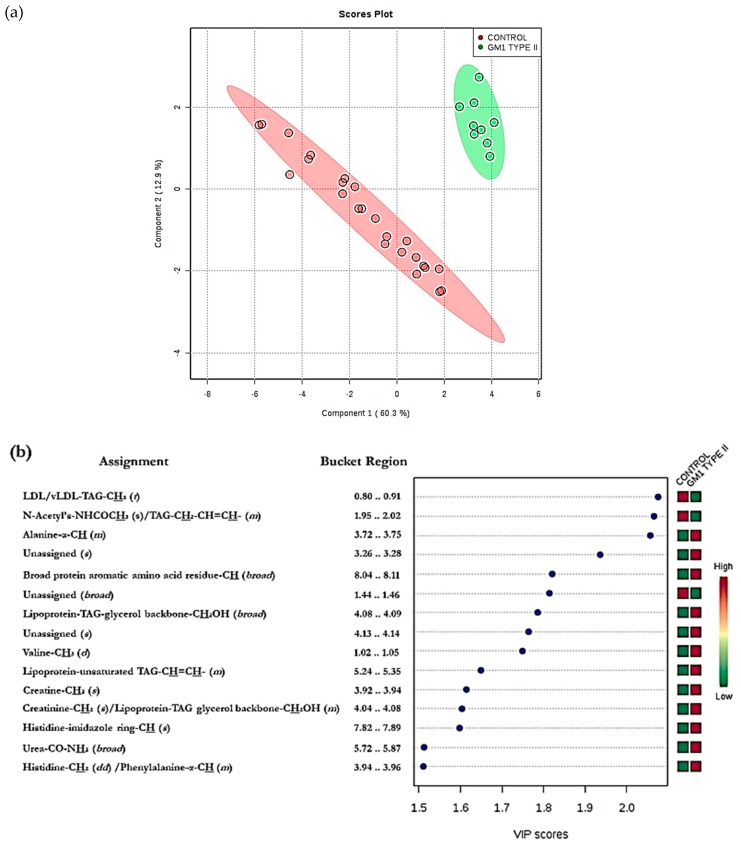
Multivariate analysis of spectral data from GM1 Type II and control plasma integral bucket regions. (**a**) PLS-DA scores plot showing controls (red) and GM1 Type II (green) together with 95% confidence ellipses. PC1 and PC2 represent 60 and 13% of the dataset variance respectively. Removal of all glucose ISB regions, which were clearly elevated in GM1 Type II participants in view of the non-fasting of participants, was performed prior to simulation of this plot. (**b**) Plot of variable importance parameter (VIP) values arising from the PLS-DA analysis, showing significantly greater intensity intelligent bucket regions, with their corresponding metabolite assignments. The red and green right-hand side abscissa axis represent metabolites which are elevated or depleted in GM1 Type II or control plasma samples respectively. The higher the VIP score, the more important the bucket region is considered in the PLS-DA model (all VIP values ≥ 1 are considered significant). Abbreviations: LDL, low-density-lipoprotein; TAG, triacylglycerol; vLDL, very-low-density-lipoprotein.

MV metabolomics analysis using ^1^H NMR data by PLS-DA revealed a clear distinction between the two disease classification groups (i.e., GM1 type II (juvenile onset) gangliosidosis versus. healthy controls, (Figure 3)). Permutation testing demonstrated a high level of discrimination between these two classifications (*p* = 0.0015). Variable importance parameter (VIP) values also confirmed a high level of significant differences between many of the ^1^H NMR intelligently selected bucket (ISB) variables; for example, downregulated lipoprotein-associated triacylglycerols in the GM1 type II gangliosidosis patients, observations which indicate overall perturbances in lipid metabolism in this disease (Figure 2). 

This observation is of particular interest, since the GM1 Type II participants were unfasted while the control ones were fasted, and therefore we would expect an elevation in lipids in the GM1 Type II participants. The top five most significant metabolites in the VIP bucket regions included 0.80–0.91 (very-low-density-lipoprotein (vLDL) and low-density-lipoprotein (LDL) triacylglyerol (TAG)-CH_3_) 1.95 – 2.02 (*N*-acetyl-CH_3_/TAG-CH_2_-CH=CH- signal), 3.72 – 3.75 (Unassigned), 8.04 – 8.11 (trigonelline) and 1.44 – 1.46 ppm (unassigned (*d*)) (Figure 3). The most significant bucket found from this MV analysis was attributable to vLDL/LDL-TAGs; however, these significant metabolites may arise from age differences between GM1 Type II and the control participants, i.e., the GM1 Type II patients are younger with mean ± SEM ages of 11 ± 2.3 years and 22 ± 0.7 years for GM1 Type II and controls respectively. This pilot investigation highlights the importance of ensuring that such metabolomics studies recruit participants who are age-matched as much as possible. However, we would expect plasma creatinine levels to rise with increasing age, but the reverse effect was observed with GM1 Type II patients, which represent the younger cohort. Creatinine is upregulated in GM1 Type II patients, an observation ascribable to muscle weakness; alternatively, these increases may arise from dietary sources [79]. Histidine, trigonelline and valine were also elevated in GM1 Type II patients, but this elevated concentration is likely to arise from dietary sources and the different fasting status of the participants involved in this investigation [79,80]. However, elevated blood histidine has been correlated with ataxia, psychosis, and tremors [79], the first of which (ataxia) is symptomatic of the GM1 Type II condition. Sample storage length could also impact on metabolite concentrations in the samples investigated, and this correlation has been demonstrated elsewhere [81]. Samples analysed over a 2-year period demonstrated that metabolites present in the blood serum of fasting individuals are more stable, and correspondingly, more pronounced in unfasted serum samples [81]. Modifications to many metabolites were observed, significant ones including phosphatidylcholines, acylcarnitines and sphingolipids, which were upregulated in fasting serum samples versus unfasted serum samples [81]. Taurine was also observed to be significantly elevated [81]. Such modifications clearly complicate any metabolomics investigations, and hence rigorous control experiments are required in these circumstances. Therefore, we recommend a combination of univariate and multivariate analyses as the best approach. Sample storage could also partially influence the results acquired here, since the GM1 type II samples were collected in the 2011–2017 period, and hence were stored for longer than the control samples, which were all collected in 2017. 

Interestingly, *N*-acetyl signals located within the δ = 1.95 – 2.01 ppm range did not appear to be markedly elevated in this biofluid, but this observation is not directly comparable to the findings made in [52], since the low-molecular-mass *N*-acetylated saccharide derivatives monitored by them were detected in the urinary profiles of these patients. Indeed, the results acquired here clearly demonstrate the complexity of metabolomics datasets, and also the importance of establishing a rigorous and robust experimental design prior to analysis. Additionally, this pilot study highlights the importance of considering all factors, including of age, diet and xenobiotics, for correct interpretation of the results. Although combinations of a series of potential biomarkers in the form of a disturbed metabolic ‘signature’ or pattern offers major advantages over the use of only a single, targeted biomarker for diagnostic purposes, reliable and successful validation of the discriminatory biomolecules found here will be best achieved by the multicomponent ^1^H NMR analysis of additional samples collected from rare GM1 Type II patients, and also an improved participant age range matching for the heathy control specimens.

A unique strategy included observing all the lipidoses simultaneously using an LC-MS-MS investigation into plasma hexosylsphingosine, globotriaosylsphingosine, lysosphingomyelin and lysosphingomyelin-509 levels [82]. This investigation observed patients with FD (*n* = 16), GD (*n* = 8), KD (*n* = 3), NPC (*n* = 11), control adults (*n* = 108) and control children (*n* = 86). From these analyses, researchers were able to distinguish these participants on the basis of these marker levels, and they found elevated globotriaosylsphingosine in FD, elevated hexosylsphingosine in GD and KD, and raised lysosphingomyelin and lysosphingomyelin-509 levels in NPC [82]. This investigation provided reference levels for the diseases, and it demonstrated the AUROC approach to be extremely sensitive and specific, with AUC > 0.98 in all cases, using 95% confidence intervals [82].

## 3. Future Prospects and Potential Limitations of Metabolomics for Monitoring of LSDs

Obtaining a sufficiently powered set of biofluid/biopsy samples is highly challenging in the case of rare diseases. However, animal trials and smaller human clinical studies can be conducted, and these have the potential to provide valuable biomarker information. Most patients are prescribed a ‘cocktail’ of different medications for symptomatic management or to slow disease progression, which can prove to be problematical for metabolomic analysis, since this mixture of medications could interfere with metabolite determinations, or alternatively mask characteristic metabolite signatures. In ^1^H NMR spectroscopy, these metabolites can be assigned and effectively removed since this technique will simultaneously detect drugs and their metabolites, in addition to endogenous biomolecules. Furthermore, in principle the effectiveness of these drugs, and their metabolism, could be simultaneously monitored. Alternatively, LC-MS strategies may provide some advantages, since a targeted metabolomics approach can be performed in view of the higher sensitivity of this technique, i.e., observation of specific metabolites that have accumulated in each disease process. There is; however, a clear lack of global, untargeted metabolomics approaches at present, with only a small number of studies [16,17] justifying NMR-based metabolomics approaches. As discussed in Ref. [83], it is not simply the specific metabolite accumulation that can be monitored, but the presence of metabolites that are not usually detectable in biofluids, and/or a reduction in the levels of metabolites which may not be critically considered in the study of LSDs. For example, the role of BCAAs in NPC diseases was not recognised until more global metabolomics approaches were applied. This global approach is slowly but surely developing, and use of a combination of bioanalytical techniques is hence becoming more common [84]. Furthermore, although the overall statistical power of MV analysis strategies is greater than those of univariate ones, the majority of researchers involved in this area also apply to the latter. Indeed, combining univariate and MV analyses is highly recommended.

It is important to note that it is possible for a variable to be simultaneously statistically significant in a univariate analysis context, and concomitantly not be ranked within the top variables of MV models applied. Indeed, initial MV analysis strategies make use of **all** variables simultaneously, and therefore they focus on simultaneous relationships (linear or otherwise) among all those available. Indeed, these approaches are based on covariances/correlations between these variables, i.e., they reflect the extent of these relationships between potentially large or very large numbers of variables, unlike univariate methods which simply consider the mean and variance values of single ones. Therefore, it is perfectly feasible that a variable which is significant in a univariate context may also be insignificant in a MV testing model. 

However, in multidimensional metabolomics investigations, the observation of a univariately significant variable which is also insignificant when tested in MV models is explicable by the ‘masking’ of information by relatively large numbers of uninformative variables. The difficulties that arise are associated with the provision of reliable estimates of covariances (notably when there are large numbers of potential predictor variables and relatively smaller sample sizes), and/or a lack of overlap between the univariate and MV testing approaches employed [85]. 

Conversely, the observation of a multivariately significant variable that is not significant if subjected to one or more univariate tests is a relatively common occurrence. Indeed, such observations are explicable by: 

(1) The complementation of independent predictor variables, i.e., separately they **do not**, but when considered together they **do** serve to explain differences observed between two or more disease classifications (e.g., LSD versus age-matched healthy controls) for comparison; 

(2) A consistency effect, i.e., in univariate statistics, although group means of single metabolite concentrations may differ between classifications, no overall significant difference may be observed in view of high levels of biological and/or measurement variation. However, with the employment of more than one independent metabolite variable to explain such classifications, both biological and measurement variations may be averaged out by the conversion of these variables to linear combinations of them, for example, as principal components (PCs) in Principal Component Analysis (PCA); and

(3) Although less relevant to examples in which there are large sample sizes (for example, > 400), and relatively low numbers of potential ‘predictor’ variables (say ≤ 20), corrections applied for multiple univariate testings, such as that of Bonferonni, or the milder false discovery rate (FDR) one, unfortunately enhance the risk of false negatives: a false negative, or Type II error occurs when the null hypothesis is actually false, but the test performed on it fails to reject it. 

Applying metabolomics to LSDs may serve to facilitate early stage disease diagnosis by providing biomarkers which serve to enhance our understanding of them at the biochemical and cellular levels. Metabolomics performed on appropriate cell extracts and cellular culture media could be performed using ^1^H NMR analysis, and intact (live) cells can also be analysed using high-resolution magic angle spinning (MAS) ^1^H NMR techniques both pre- and post-gene inactivation. This may provide biomarkers and it may also provide information regarding potential drug targets, together with further understandings of the disease mechanisms, as recently shown by [59]. Earlier detection could give a more promising outlook, including an earlier course of gene therapy treatment prior to disease manifestations taking effect.

Moreover, few of the above studies noted in Table 2 have applied power analysis as a tool to estimate the number of participants/patients required to be recruited in order to satisfy statistical significance to a level which is reproducible, in a univariate, multivariate or both contexts. Since the number of participants undertaking such studies are relatively small, or in some cases extremely limited (e.g., [11,50,52]), the significance of the stratification and application of results acquired could be overestimated. Both type I (false positive) and type II (false negative) errors can occur; the latter error can be evaluated by applying power analysis calculations in order to assess the sensitivity of the statistical test system [8]. Statistical power can be influenced by three factors inclusive of: (i) degree of the effect of the monitored metabolite, (ii) the statistical conditions set, and (iii) the sample size [86]. Both the sample size and the statistical condition set are defined by the analyst prior to data collection. Power calculations are essential and generally beyond reproach, since their application ensures that there is sufficient evidence to support a successful metabolomics investigation and data acquired has reproducibility (precision) [8]. Equally, if an excess of samples is acquired, an overspend on resources would occur and this is a hindrance from an economic perspective, in addition to the ethical implications, such as exposing a surplus of participants to selected risk factors/hazards [8,87]. 

Power analysis methods include *MetSizeR* and *Data-driven Sample Size Determination* (DSD) [86]. DSD is an algorithm which calculates the sample size required to identify at least one statistically significant variable or a maximum number of such variables. The statistical recoupling of variables (SRV) algorithm is applied first to enable data reduction prior to analysis and to focus on metabolically significant biomolecules, followed by computing inverse cumulative density probabilities (ICDP) [86]. Kernel density estimate function or log normal distribution fitting are applied for each ICDP. An expanded dataset is then simulated at different sample sizes, since random numbers are drawn in the ]0, 1[ interval and as input quantiles of the ICDPs [86]. Statistical significance is then evaluated using analysis of variance (ANOVA), and false discovery rate (FDR) values are measured using the Benjamini-Yekutieli correction [86]. DSD produces a table with mean and SD values of statistically significant variables for each dataset size. Based on PCA, *MetSizeR* does not apply a data reduction strategy, whereas DSD uses the orthogonal partial least squares-discriminatory analysis (O-PLS-DA) approach, and therefore the above SRV values are tested. However, *MetSizeR* is a targeted approach, since it can only cope with a maximum of 375 variables, forcing the user to pre-select these; however, DSD does not place any restrictions on the number of variables incorporated. Standard parameters are specified in the *MetSizeR* guidebook [88]. This investigation used an experimental dataset of rat urine (*n* = 36) in two equivalent treatment groups collected longitudinally over a 15-day period analysed by ^1^H NMR [88]. Both power analysis approaches confirmed that fewer than 30 samples were required for optimum sample size in the experimental dataset of rat urine. 20 and 30 samples were determined by the DSD and *MetSizeR* strategies, respectively, and hence these appeared to be reliable methodologies for assessing the size of the experimental dataset. Other methodologies include use of SSPA from the *Bioconducter R* package [89,90], which have previously been reviewed [8]. 

**Figure 4:**
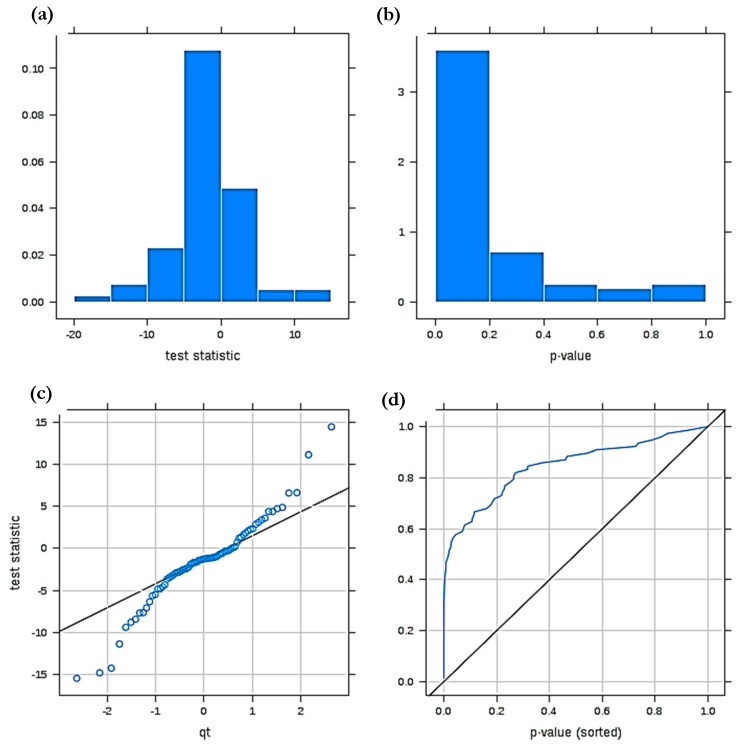
Diagnostic plots derived from a MV statistical power calculation conducted on a model involving comparisons between the ^1^H NMR profiles of human blood plasma collected from healthy humans (controls) and GM1 type II gangliosidosis patients. (**a**) t statistic value *p* value distribution displayed as a histogram; (**b**) corresponding QQ plot of these *t*-test values (i.e., t versus qt value); (**c**) left-shifted distribution of t-test statistic *p* values for this power analysis; (**d**) plot of sorted t statistic *p* values versus their ranking. The dataset was constant-sum normalised, cube root-transformed and Pareto-scaled prior to analysis.

Figure 4 shows diagnostic plots arising from a power calculation performed on the above pilot GM1 type 2 gangliosidosis dataset analysed by *MetaboAnalyst 4.0*, which arises from the *Bioconductor R* package SSPA. This approach estimates the mean MV analysis power level for selected FDRs and was originally developed for estimating sampling sizes for pilot gene expression profiling datasets [89]. These diagnostic plots allow us first to evaluate the normality of two sample *t* test statistic values employed to prime this system, and second the availability of significant ‘between-disease classification’ mean differences for our pilot ^1^H NMR ISB dataset consisting of 37 samples and 83 ISBs. For this pilot dataset, Figure 4a shows that the t statistic value histogram obtained does not appear to deviate significantly from normality, as does the QQ plot shown in Figure 4b. Additionally, Figure 4c displays the distribution of two-sample t-test statistic *p* values, also as a histogram, confirming that a large fraction of these lie within the 0.00–0.20 range, i.e., it is left-shifted, and this distribution provides strong evidence for a large number of significant ‘between-disease classification’ variables among the 83 ISBs tested. A plot of sorted *p* values against their rankings (Figure 4d), confirms a high degree of statistical discrimination between these classifications for the ISBs considered, i.e., this plot is markedly shifted towards the top left-hand corner. 

**Figure 5:**
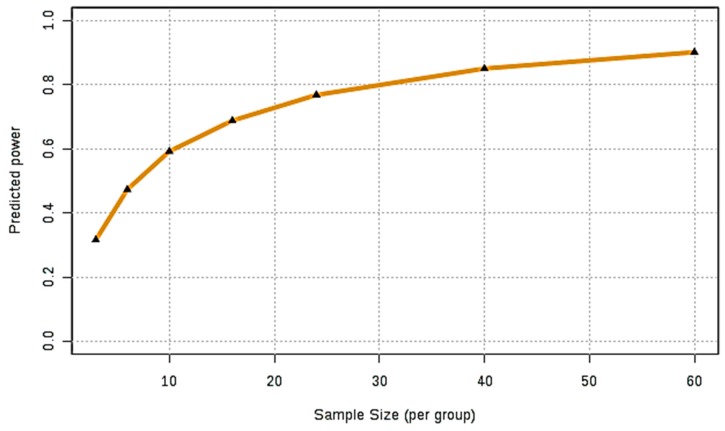
Power analysis of GM1 Type II and controls. Predicted power curve for our pilot GM1 type II disease ^1^H NMR profile dataset containing *n* = 28 healthy controls and only 9 with this LSD. The FDR *p* value for this power calculation was pre-set at a value of 0.05, and normalisation, transformation and scaling were performed as described in Figure 3 legend.

Once a high degree of statistical certainty was ascertained, the power calculation was performed, and Figure 5 depicts the predicted power curve based on the pilot dataset investigated, i.e., a plot of predicted power against sample size requirement per classification. This curve shows that for an FDR value of 0.05, a sample size approaching *n* = 30 per disease classification test group is required for a satisfactory model with the ability to clearly distinguish between these, specifically one with an overall power of 0.80. Although for our pilot blood plasma dataset this value was very nearly realised for the healthy control group (*n* = 28), that for the gangliosidosis condition was only *n* = 9. However, limited numbers of patient participants, and collected biofluid sample volumes, which successfully match those of an age-matched healthy control population, are unfortunately rather commonplace in the field of LSDs in view of their rarity. 

Although the authors of Ref. [91] recently discussed some future perspectives for metabolomics in terms of inborn errors of metabolism, these considerations could be further developed. For example, a thorough metabolomics analysis of the effects of ERTs and SRTs on patients with LSDs could provide vital insights into the effectiveness of these clinical approaches by observing biomarkers in a multidimensional context, a simple example being the simultaneous observation of elevated markers decreasing, and depleted markers rising in their biofluid concentrations. New robust, non-invasive treatment options need to be explored for LSDs. In the case of neurological disorders, variants of these therapies are required to successfully cross the BBB. To enhance ERT, the use of nanoparticles to facilitate enzyme crossing the BBB is also being explored, since the nanoparticles protect the enzyme delivered against its in vivo degradation [92]. Thus, metabolomics could play a role in ensuring that this technology is effective and perhaps also serve to validate candidate biomarkers. Moreover, nanoparticles are also being employed for the gene therapy trialling of non-viral vectors, and again metabolomics could provide insightful information regarding the effectiveness of such therapies [92]. However, one major potential drawback of nanoparticles is that their long-term toxicity remains unknown, a feature which again could be explored using metabolomics as a vital probing tool. 

Urine samples are yet to be analysed to the same extent as blood plasma/serum and CSF in these patients, and donations of this biofluid obviously constitutes a less invasive collection procedure. Although all biofluids and tissues have been probed in these works, plasma appears to be the biofluid of choice for such studies. Interestingly, no metabolomics study has been performed on human saliva. Such a study would be advantageous for virtually non-invasive sampling purposes. In principle, saliva also has the potential to represent the full physiological status of the human body, just as blood serum or plasma samples can. It should be noted that many fully characterised, established blood plasma markers are often present in saliva at much lower concentrations [70]. Salivary biomarkers have been found using metabolomics in diseases such as cancer and diabetes [70]. However, the disadvantage of saliva for such investigations is that it may easily be compromised, i.e., influenced by external factors such as poor oral health/hygiene, and eating or drinking episodes prior to sampling, Hence, these investigations should be carried out with extreme care, particularly careful sample collection [93]. Moreover, very few studies have been performed on brain tissue from living participants in view of the inability to retrieve such samples. However, murine brain has been analysed as described above [15,54,72]. However, several biofluids, tissues and cells that can be investigated for LSDs, e.g., urine and CSF in KD remain unexplored. NPC and FD have been investigated by MS- and NMR-linked metabolomics investigations thoroughly, with a total of 24 and 17 publications arising from the study of these diseases, respectively. Less metabolomics research has been performed on KD and GD, however, with only four and five publications targeting each disease, respectively at the time of this report, and very little has been completed on gangliosidoses. These are areas which could be further investigated. It would also be interesting to explore metabolic correlations of both biomarker and non-biomarker variables between differing sub-classes of the same disorder, i.e., NPA, NPB and NPC, and any similarities they may have with GD and gangliosidosis. Metabolic correlations have shown to be extremely useful in other investigations to establish the specificity of the marker [82].

Lack of uniform studies across all LSDs is evident within this review in terms of experimental design and statistical approach, not to mention the bioanalytical strategies and techniques employed for these purposes. More systematic metabolomics studies will provide a wealth of information regarding the significance of the current biomarkers identified, and also enable expedient cross-validation of metabolites. Larger scale studies are also required, since they aim to provide many more representative dataset profiles. The study of Reference [27] is a good example of this. As discussed above, it is also important to use a biomarker or combination of biomarkers which are rigorous enough to avoid misdiagnosis, as demonstrated by Ref. [26,28]. 

LC-MS-MS shows promise for the detection of NPC disease, demonstrating an elevated level of blood plasma cholestane-3β5α6β-triol. However, there is a need for such developments to be integrated into clinical practice, perhaps in combination with one or more specific biomarker for potentially confounding sterol disorders, or for NPC disease itself. A combination of the phenome, metabolome, and genome could translate the research in metabolomics into practice to modernise medicine; however, for metabolomic applications to operate effectively, method development and validation are also crucial [4]. One of the major disadvantages of translating MV NMR datasets into medical practices is that the spectroscopic instruments, such as NMR and LC-MS, have both high purchase and maintenance costs, and require specialist training for use, interpretation and subsequent data analysis. However, NMR technology is also showing some promise in benchtop instrumentation, such that it could be relevant for ‘point-of-care’ clinical applications, especially with the advent and development of water suppression sequences and automation now possible [94]. NMR approaches may prove to be invaluable as substantial technological advancements are being made in this field, with technologies such as hyperpolarisation increasing instrumental sensitivities. Computational tools are becoming an integral part of metabolomics applications, and artificial intelligence technology could effectively simplify and distinguish normal from unusual patterns of results acquired by metabolomics-based instrumentation. 

Another area of metabolomics research which is yet to be explored in depth is neonatal and prenatal testing for LSDs. Some investigations have been performed on paediatric patients, and to date this testing protocol offers some opportunities for further developments within this field [33,95]. These diseases are often overlooked in view of their rarity, and hence diagnosis and prognosis are not established until later in the lifespan of the patient. Notwithstanding, currently available reports have emphasised that early detection of these diseases is a highly beneficial patient requirement, and hence early testing and diagnosis could indeed represent a life-saving process. 

The impact of multivariate metabolomics analysis in healthcare, particularly for diagnostic purposes, offers much potential for lysosomal storage disorders. Indeed, for these disorders, available literature has revealed the availability of multiple clinical biomarkers, which may provide much valuable information regarding the status of an array of metabolic pathway disturbances simultaneously. Its advantages over other diagnostic tools, such as genetics or enzymatic probes, is that such multianalyte approaches are able to provide a virtually complete metabolic ‘snapshot’ of the pathological status of living systems at particular points in time. Although this is a valuable approach for longitudinal prognostic or therapeutic intervention studies, the authors thoroughly recommend the repeated collection of two or more biofluid samples within narrow or longer diurnal timepoints for diagnostic purposes in order to evaluate the repeatability of profiles acquired. Moreover, all possible steps to avoid potential interferences arising from demographical or other covariables should be taken. 

Hence, genomic and metabolomic assessments, along with appropriate enzyme assays are considered complimentary to each other. In turn, carefully designed metabolomics approaches can provide insights into treatment outcomes, successful or otherwise. Therefore, combining a metabolomics probes with other tests, such as the assessment of cognitive and organ functions, would provide an improved overall patient overview. Indeed, established and carefully validated biomarkers can be simultaneously informative in diagnosis, prognosis, and treatment options, and could therefore provide much improved outcomes for patients by developing a more robust understanding of the molecular, cellular and tissue networks involved concomitantly. This removes reliance on symptomatic networks, and moves towards the more pro-active diagnosis of patients, rather than ‘reactive’ diagnosis which is-based solely on patient symptoms.

In summary, despite some progress, metabolomics approaches have yet to influence diagnosis and monitoring of LSDs in clinical practice. Clinical diagnosis is dominated by PCR-based genetic testing and enzyme assays, and hence using metabolomics to diagnose LSDs is still in its infancy. Nevertheless, future opportunities in this area remain, especially in an era in which personalised medicine is becoming more common. Future developments required for LSDs include (1) systematic sample collection and handling; (2) further validation of at least some of the potential biomarkers suggested via the rigorous monitoring of their biofluid levels along with simultaneous co-ordinated assessments of clinical responses to treatment(s); (3) detailed correlations of biomarker concentrations with disease severity, and also demographic variables such as age, gender and diet, etc.; and (4) a further detailed review in order to ensure that biomarker(s) identified are solely specific to particular LSDs. 

## 4. Conclusions

The multicomponent analysis of biofluids, tissues, cells and/or corresponding cell culture media using high-resolution NMR, LC-MS, and LC-MS-MS platforms, when coupled with MV data analysis techniques, is an extremely powerful means of probing the biochemical basis of human disease aetiologies, such as those featured in LSDs. This strategy clearly offers major research advantages, especially in view of the relatively limited amount of biomolecular information derivable from the direct computer-visual examination of multicomponent spectral or chromatographic profiles acquired (including the determination of many individual biomolecule levels) by trained, expert staff, a process which is extremely costly, labour-intensive and time-consuming.

To date, metabolomics techniques have only scratched the surface in terms of their applications to the lipidoses; however, they have provided some promising diagnostic and prognostic information for NPC1 disease. Similar, more extensive investigations could be performed for other LSDs such as GD, KD and Gangliosidoses. In addition, more global experimental design and bioanalysis strategies may provide more valuable information, rather than limiting research to that focused only on a limited number of biomarkers as in pre-targeted methodologies. However, such metabolomics-based investigations remain somewhat limited in view of the limited number of biofluid or solid biopsy samples available from humans suffering from these debilitating conditions. Although this is not a problem experienced with animal models of LSDs. Therefore, the authors thoroughly recommend the performance of MV power analysis of pilot data prior to the design of experiments for these diseases, as demonstrated here in Figure 3 and Figure 4. Additionally, carefully planned meta-analysis studies involving MV adjustments may also offer support for both targeted and untargeted LSD biomarker research. 

## Figures and Tables

**Table 1:** Gene defects and approved therapies for lipid storage disorders.

Lipid Storage Disorder	Defected Gene	Approved Therapies
GM1 Gangliosidosis	*GLB1*	S/S
Sandhoff Disease (GM2)	*HEXB*	S/S
Tay-Sachs Disease (GM2)	*HEXA*	S/S
Niemann-Pick diseases type A & B (NPA & NPB)	*SMPD1*	S/S
Niemann-Pick disease type C1 (NPC1)	*NPC1* (95%)	SRT
Niemann-Pick disease type C2 (NPC2)	*NPC2* (5%)	SRT
Gaucher Disease (GD) types I-III	*GBA*	ERT and SRT
Fabry Disease (FD)	*GLA*	ERT and CT
Krabbe Disease (KD)	*GLB*	HSCT and S/S

Sphingolipidoses with corresponding defective genes and approved therapies. Abbreviations: CT, Chaperone Therapy; ERT, Enzyme Replacement Therapy; HSCT, Haematopoietic Stem Cell Therapy; S/S, Symptomatic and Supportive Therapy; SRT, Substrate Reduction Therapy.

**Table 2:** Biomarkers for lipid storage disorders.

Disease	Sample Type	Methodology	Sample Size	Potential Biomarkers Upregulated (↑) or Downregulated (↓)	Statistical Models and Analysis	References
(1) NPC	Mouse Brain Mouse Liver Mouse Spleen	LC-MS-MS	~20 specimens	↑ Sphingolipid species in the liver ↑ GM2 and GM3 gangliosides in the brain and spleen	Results expressed as mean±SEM values; two-tailed single factor ANOVA model (*p* ≤ 0.05 was considered significant). Bonferroni correction applied. Biomarker heatmap.	[9]
	Human Plasma Human CSF	LC-MS-MS	56 NPC1 56 Controls	↑ Monohexosylceramide and ceramide ↓ Sphingoid bases		
	Human Plasma	LC-MS-MS	109 NPC1 88 Controls 45 Heterozygous carriers	↑ 7-Ketocholestrol and cholestane-3β5α6β-triol	Results expressed as mean ±SEM values; two-tailed single factor ANOVA model (*p* ≤ 0.05 was considered significant).	[10]
	Human Plasma	LC-MS-MS	5 NPC1 7 Controls	↑ Lysophingomyelin-509 and sphingosylphosphorylcholine	Results expressed as mean ± SD; Student’s t-test	[11]
	Human Plasma	LC-ESI-MS-MS	13 NPC1 60 Controls	↑ Cholestane-3β5α6β-triol and 7-ketocholestrol	Preliminary Kolmogorov-Smirnov test. Student’s t-test for normally distributed variables and a Mann-Whitney test for non-normally distributed variables. Pearson correlation coefficients were employed to evaluate relationships (*p* < 0.05 was considered significant). ROC curves were generated (0.998 for cholestane-3β5α6β-triol): specificity of 98.3%; sensitivity of 100%.	[12]
	Human Plasma	LC-MS-MS	148 non-NPC1 6 NPC 11 NPB1 NPA	↑ Cholestane-3β5α6β-triol	Box and whisker plot, mean values only.	[13]
	Human Urine	LC-ESI-MS	1 NPC1 patient 1 3β-HSD deficiency 1 control infant 1 control adolescent	↑ Sulphate conjugates of cholesterol and bile acids in NPC and 3β-HSD deficiency	Counts reported in NPC and 3 β-HSD and no counts reported in controls. No statistical analysis performed.	[14]
	Human Plasma	GC-MS	25 fasting NPC1 25 Controls 23 heterozygotes	↑ Cholestane-3β5α6β-triol and 7-ketocholestrol	Results expressed as mean±SEM values. Two-tail single factor ANOVA (*p* ≤ 0.05 was considered significant). Bonferroni correction applied. Cholestane-3β5α6β-triol (*p* < 0.001) ROC curve AUROC value 1.0. 7-ketocholesterol (*p* = 0.05) ROC Curve AUROC value 0.9984.	[15]
	Mouse Liver	^1^H NMR	28 Wild-Type 31 Heterozygote 30 NPC1	↑ Phenylalanine, tyrosine glutamate, lysine/ornithine, valine threonine, hypotaurine/methionine ↓ Inosine nicotinate/niacinamide, phosphoenolpyruvate, 3-hydryphenylacetate	Analysis of covariance (ANCOVA) model incorporating 3 first-order interaction components of variance; ANOVA-simultaneous component analysis (ASCA); Random Forest (RF) model (out-of-the-bag error value 0.175 and 0.19); AUROC value 0.94; Metabolic pathway topological analysis (MPTA).	[16]
	Human Plasma	^1^H NMR	40 untreated NPC Total 89 from 34 in duplicate treated with Miglustat over 2-year period 30 Control 31 Heterozygote	↓ HDL-cholesterol and LDL-cholesterol in NPC vs Control ↑ Triacylglycerol in NPC vs Control ↑ Ca^2+^ ions in NPC vs HET ↓ HDL-Cholesterol in NPC vs HET	ANOVA with Bonferroni correction. PCA scores plots (RF model: Out-of-the-bag error 0.089±0.0002 (mean±SEM) Canonical correlation analysis (CCorA).	[17]
	Human Urine	LC-ESI-MS-MS	2 NPC patients 2 3B-hydroxysteroid dehydrogenase deficient patient 8 controls	↑ 3β-Sulfooxy-7β-*N*-acetylglucosaminyl-5-cholen-24-oic acid, and its glycine-amide and taurine-amide derivatives	Mean±SD values only.	[18]
	Human Urine	LC-MS-MS	23 NPC patients 28 Controls 7 Patients with cerebrotendienous xanthomatosis, glycogen storage disease types I and II, citrin deficiency and abetalipoproteinemia	↑ 3β-Sulfooxy-7β-*N*-acetylglucosaminyl-5-cholen-24-oic acid, and its glycine-amide and taurine-amide derivatives	Wilcoxon’s t-test applied to all metabolites detectable (*p* < 0.05) AUROC value > 0.95 for each marker	[19]
	Human Urine	LC-MS-MS	28 NPC Patients 38 Controls	↑ 3β-Sulfooxy-7β-hydroxy-5-cholen-24-oic acid, 3β-sulfooxy-7-oxo-5-cholen-24-oic acid, non-amidated 3βsulfooxy-7β-*N*-acetylglucosaminyl-5-cholen-24-oic acid, glycine-amidated 3β-sulfooxy-7β-*N*-acetylglucosaminyl-5-cholen-24-oic acid and taurine-amidated 3β-sulfooxy-7β-*N*-acetylglucosaminyl-5-cholen-24-oic acid	AUROC value > 0.92 for each marker AUROC value 1.0 for 3β-sulfooxy-7β-*N*-acetylglucosaminyl-5-cholen-24-oic acid	[20]
	Human Plasma	HPLC-MS-MS	135 NPC1 Patients 66 NPC1 Carriers 241 Other LSD 46 Controls	↑ Cholestane-3β,5α,6β-triol and lyso-Sphingomyelin-509	ROC (100% sensitivity and 91% specificity) and AUROC value 0.99 with 95% CI: 0.98–1.00 ANOVA (*p* < 0.05).	[21]
	Human Urine	^1^H NMR	13 untreated NPC1 47 control heterozygote carriers	↑ Bile acids, BCAAs, 3-aminoisobutyrate, glutamine, 3-methylhistidine, creatine, quinolinate, succinate, trimethylamine, nicotinate, *N*-methylnicotinamide, *N*-methyl-2-pyridone-5-carboxamide, *N*- methyl-4-pyridone-5-carboxamide and trigonelline	Cube root-transformed, Pareto-scaling, false discovery rate or Holm step-down Bonferroni correction; normalisation to Cn; ANOVA; Principal component analysis (PCA); Partial least-squares discriminatory analysis (PLS-DA): Q^2^ = 0.56 and Accuracy 0.93 (*p* = 5.0 × 10^−4^); Partial redundancy analysis (P-RDA) *p* < 10^−4^; MV ROC AUROC value 0.93 (95% CI 0.78–0.99). UV ROC AUROC value 0.81–0.90; RF model 83% overall mean classification success; Heatmap generated.	[22]
	Human Urine	LC-ESI-MS-MS	1 NPC	↑ 3β-Sulfooxy-7β-hydroxy-5-cholen-24-oic acid and 3β-sulfooxy-7-oxo-5-cholen-24-oic acid	No statistical analysis performed.	[23]
	Human Plasma	LC-MS-MS	70 Control 57 NPC	↑Lysosophingomyelin and glucosylsphingosine	AUC AUROC values±95% CI Lysosophingomyelin 0.9994, glucosylsphingosine 0.7764.	[24]
	Human Dried Blood Spot	LC-MS-MS	27 NPB 20 Control	↑ Lysosophingomyelin	Mean (*p* < 0.0001), Student’s t-test.	[25]
	Human Plasma	LC-ESI-MS-MS	38 NPD not ASM Deficient 7 NPD ASM Deficient 7 NPC 34 GD 12 KD >300 Controls	↑ 7-Ketocholestrol in NPC and NPB ASM deficient patients	Mean ± SEM; ANOVA (*p* ≤ 0.05).	[26]
	Human Dried Blood Spot Human Urine	UPLC-MS-MS	DBS 73 NPC1 2 NPC1 Homozygous 2 NPC1 Heterozygous 2 NPC1 Wild Type 84 Controls 70 NPC1 Heterozygotes Urine 14 NPC1 2 NPC1 Homozygous 10 Controls 47 NPC1 Heterozygotes	↑ 3β-Hydroxy,7β-*N*-acetylglucosaminyl-5-cholenoic acid 40 times higher in NPC disease than that ofcontrols; however, these values were considered normal in some patients in view of a mutation ↑ 3β,5α,6β-Trihydroxycholanoyl-glycine 10 times higher in NPC compared to controls better biomarker in dried blood spots	Kruskal-Wallis test; Dunn’s significance level of 0.05. ↑ 3β,5α,6β-Trihydroxycholanoyl-glycine (*p* < 0.001) when compared to controls and carriers in dried blood spots.	[27]
	Human EDTA Plasma	HPLC-ESI-MS-MS	107 Controls 16 NPC 91 Other diseases	7-Ketocholestrol (7-KC) unspecific biomarker ↑ Cholestane-3β,5α,6β-triol (CT) sensitive but not specific	Mann-Whitney U-test, Spearman’s correlation analysis, ROC Curve AUROC value (*p* < 0.05 considered statistically significant). NPC *vs* Controls 95% CI for CT, 62–275 vs. 3.5–4.4 ng/mL, *p*< 0.0001; 95% CI for 7-KC, 178–795 vs. 11.8–14 ng/mL, *p* < 0.0001. 100% sensitivity and 88.7% specificity.	[28]
(2) Fabry	Human Urine/Human Plasma	UPLC-ESI-MS-MS	111 Urine 129 Plasma	↑ Sphingolipids significantly in 31 plasma and 26 urine samples. 48% elevation in plasma, 42% in urine over those of controls. Phospholipids also reported to be higher: fold-changes 15% in plasma and 13 in urine.	PCA plot: Fabry patients loaded positively on PC1, Controls loaded negatively on PC1. Sphingolipids also loaded strongly on PC1.	[29]
	Human Urine	qTOF-MS	63 Untreated Fabry 59 Controls	↑ Globtriaosylceramide and globotriosylsphingosine	PCA, OPLS-DA and S-plot: *p* = 0.05.	[30]
	Human Urine	LC-MS	42 FD 48 Controls	↑ Lyso-Gb3 plus its analogues	AUROC value 1.0	[31]
	Human Plasma	LC-MS-MS	38 FD male and female patients 120 controls	↑ Lyso-Gb3	Mean ± SD or median. Whitney U test. ROC curve analysis (*p* < 0.05)	[32]
	Human Urine	qTOF-MS	16 untreated Fabry males 16 healthy Control males	↑ Galabiosyceramide Analogs	OPLS-DA, Pareto-scaling *p* = 0.05.	[33]
	Human Urine	UPLC-MS-MS	52 Fabry paediatric male 108 Fabry adults 52 control paediatric 45 control adults	Gender effects biomarkers; paediatric females have lower levels ↑ Lyso-Gb3 / related analog profile	ROC Curve AUROC values; Mann-Whitney U test; Spearman rank correlation coefficient *p* < 0.05 in Fabry male children Treated females had lower lyso-Gb3 / related analogue profiles.	[34]
	Human Plasma	Nano-LC-MS	16 Male 10 heterozygous females 5 functional variants 40 controls	Gender effects biomarkers; females have lower levels ↑ Lyso-Gb3 / related analog profile	Mean±SD Tukey multiple comparison test (*p* < 0.0001); no difference in functional variants found (p > 0.05).	[35]
	Human Plasma	UPLC-MS-MS	178 healthy controls 74 Fabry	↑ Lyso-Gb3 Lower levels in females	Specificity 100%; Sensitivity Males 95%, Females 88%; Significance Males (*p* < 0.005) Females (*p* < 0.15).	[36]
	Human Urine	LC-MS	164 Fabry 94 controls	↑ Lyso-Gb3 and analogs Lower levels in females	*p* < 0.001 lyso-Gb_3_ analogue levels in urine and gender, lyso-Gb_3_ levels analogue levels correlated well with enzyme replacement therapy (ERT) in males (*p*< 0.05). Mann-Whitney U-test and Spearman rank correlations were performed.	[37]
	Human Plasma	HPLC-ESI-MS-MS	48 untreated Fabry 79 treated Fabry 36 control	↑ Lyso-Gb3 Lower levels in females Untreated Fabry males distinguishable from controls; ERT- treated Fabry males less so.	Box-and-whisker plots only.	[30]
	Human Plasma	HPLC	10 Fabry Male 8 Heterozygote Female	↑ Lyso-Gb3 increased in males	Mean±SD values. Student’s t-test *p* < 0.05 considered significant. Males significant (*p* < 0.05)	[38]
	Human Urine	UPLC-MS-MS	150 Fabry Patients 95 Controls	↑ Gb3 Isoforms	Mann-Whitney U Test (*p* < 0.001) established significant differences between Gb3 Isoforms, genders, treatments and concentrations.	[39]
	Human Plasma Human Urine	UPLC-ESI-MS-MS	10 Fabry Hemizygotes 10 Fabry Heterozygotes 20 Controls	↑ Lyso-Gb3 in both urine and plasma	Mean±SD values only.	[40]
	Mouse Plasma Mouse Spleen Mouse Heart Mouse Liver Mouse Kidney	LC-MS-MS	Wild Type (*n* = 3) Fabry (*n* = 5)	↓ Significantly lower lyso-Gb3 after administration of 0.5 mg/kg human α-galactosidase A mRNA in plasma, spleen, heart, liver and kidney	Mean±SD/SEM and ANOVA	[41]
	Human Plasma Human Urine	LC-MS-MS	18 asymptomatic females 18 symptomatic females 27 males 27 control urine 58 control plasmas	↑ C22:1, C22:0, C22:1-OH, C22:0-OH, C24:2, C24:0, C24:2-OH, C24:1-OH, C24:0-OH, C26:0 Galabiosylceramide in asymptomatic females	PCA and OPLS-DA of urine and plasma samples. Kruskal-Wallis and Mann-Whitney tests used for univariate comparisons. ROC curve analysis.	[42]
(3) Gaucher	Human Serum Human Peritoneal Fluid Human Pericardial Fluid	MALDI TOF-MS	2 Gaucher Disease 1 Leukemia 3 Ovarian Tumors 5 Respiratory Infection	↑ Glycosylceramide in all biofluids examined for GD patients	Mean±SD values only.	[43]
	Human Plasma	HPLC	27 Gaucher Disease Type I 15 Control	↑ Glycosylceramide in disease *vs* control ↓ Glycosylceramide and Glycosylceramide /Ceramide ratio in disease *vs* 6 months of treatment with ERT/SRT group	Median and range values; Mann-Whitney U test; Correlations tested by rank correlation test (Spearman coefficient) *p* < 0.05 considered significant. Glycosylceramide *p* = 0.0327 and Glysocylcarmide/ceramide ratio *p* = 0.0034 after 6 months after receiving ERT/SRT.	[44]
	Human Plasma	LC-MS-MS	148 Controls 98 GD 13 GD Carriers 262 Patients with other LSD including NPC KD FD and Hunters Disease	↑ Glucosylsphingosine	100% specificity/100% sensitivity AUC 95% CI in ROC Analysis: AUROC value 1.0.	[45]
	Human Plasma	LC-ESI-MS-MS	64 GD 28 control	↑ Glucosylsphingosine 200-fold higher than controls Positive correlations between glucosylsphingosine and sphingolpids established in addition to plasma glucosylsphingosine and liver volume, bone marrow fat fraction	Median and range, Mann-Whitney U test, rank correlation test (Spearman coefficient) statistically significant when 2-tailed *p* < 0.05 glucosylsphingosine and sphingolipids correlation, with *p* = 0.002.	[46]
	Dried Blood Spots	LC-MS	35 Mild Type I GD 34 Severe Type I GD 12 Type III GD	↑ Glucosylsphingosine	Median and range reported, non-parametric Spearman’s and parametric Pearson’s correlations employed to observe relationships.	[47]
(4) Krabbe	Mouse Hindbrain Tissue	GC-MS/LC-MS	8 Wild Type 8 ‘Twitcher’	↑ Hypoxathinein Twitcher ↓ Glucose usage in Twitcher, phospholipid and membrane turnover in Twitcher, cholesterol lanosterol and lathosterol in Twitcher ↓ *N*-acetylaspartate	ANOVA, Random forest model Student’s t-test *p <* 0.05 considered significant *P* ≤ 0.05 12 metabolites significantly modified RF 31%-100% accuracy dependent on collection point.	[48]
	Dried Blood Spot (DBS)	LC-MS-MS	75 Controls Newborns 8 Newborns 65 Krabbe Disease	↑ Pyschosine in KD	Mean±SEM values only.	[49]
	DBS	HPLC-MS-MS	23 ‘at-risk’ KD newborns 8 KD	↑ Pyschosine in KD	Mean values only.	[50]
	DBS	LC-MS-MS	220 Controls 26 KD 18 GALC mutation	↑ Pyschosine in KD	Range of values only.	[51]
(5) G_M1_ and G_M2_	Urine	Preparative TLC-^1^H NMR Analysis	10 Controls 50 Diseased samples including GM1, GM2, Salla, Fucosidosis, Tyrosinena Type I and II, Citrullinemia, Canavan Disease, French-type sialuria, α- and β- mannsidosis, aspartylglu-cosaminuria	↑ *N*-acetylated biantennary octasaccharide, *N*-acetylated triantennary decasaccharide/ tetraantennary dodecasaccharide in GM1 ↑ Oligosaccharides in GM2	No statistical analysis performed	[52]

Suggested biomarkers from metabolomics investigations for LSDs using LC-MS or NMR-based methodologies. Abbreviations: 3β-HSD, 3β -hydroxysteroid-∆-^5^C_27_-steroid dehydrogenase; ANCOVA, analysis of covariance; ANOVA, analysis of variance; ASCA, analysis of variance simultaneous component analysis; AUC, area under the curve; AUROC, area under receiver operating characteristic; BCAA, branched chain amino acid; CCorA, canonical correlation analysis; CT, cholestane-3β,5α,6β-triol; DBS, dried blood spot; ERT, enzyme replacement therapy; ESI, electrospray ionisation; FD, fabry disease; GC, gas chromatography; GD, gaucher disease; HDL, high density lipoprotein; HET, heterozygous; HPLC, high performance liquid chromatography; KC, 7-ketocholesterol; KD, Krabbe disease; LC, liquid chromatography; LDL, low-density-lipoprotein; LSD, lysosomal storage disorder; lyso-Gb3, globotriaosylsphingosine; MALDI, matrix-assisted laser desorption/ionization; MS, mass spectrometry; MV, multivariate; NMR, nuclear magnetic resonance; NPA, Niemann-Pick Disease Type A; NPB1, Niemann-Pick Disease Type B1; NPC1, Niemann-Pick Disease Type C1; O-PLS-DA, ortho-partial least squares discriminant analysis; PCA, principal component Analysis; PLS-DA, partial least squares discriminatory analysis; P-RDA, partial redundancy analysis; QTOF-MS, quadrupole time of flight-mass spectrometry; RF, random forest; ROC, receiver operating curve; SD, standard deviation; SEM, standard error of the mean; SRT, substrate reduction therapy; TLC, thin-layer chromatography; UPLC, ultra-performance liquid chromatography.

## Supplementary Materials

Supplementary materials can be found at https://www.mdpi.com/1422-0067/21/7/2533/s1.

## Abbreviations

2,4-(S)-HC	2,4(S)-hydroxycholesterol
AUROC	Area Under Receiver Operating Characteristic
BBB	Blood-Brain Barrier
BCAA	Branched chain amino acid
CNS	Central Nervous System
CSF	Cerebrospinal Fluid
DBS	Dried Blood Spot
ERT	Enzyme Replacement Therapy
ESI	Electrospray Ionisation
FD	Fabry Disease
FDR	False discovery rate
GD	Gaucher’s Disease
HPLC-LS	High Performance Liquid Chromatography-Light Scattering
HPβCD	Hydroxypropyl-β-Cyclodextrin
ISB	Intelligent Selected Bucket
KD	Krabbe Disease
LC-MS	Liquid Chromatography-Mass Spectrometry
LDL	Low-density lipoprotein
LSD	Lysosomal Storage Disorder
Lyso-Gb_3_	Globotriaosylsphingosine
MAS	Magic Angle Spinning
MS	Mass Spectrometry
MV	Multivariate
NMR	Nuclear Magnetic Resonance
NP	Niemann-Pick Disease
NPA	Niemann-Pick Disease Type A
NPB	Niemann-Pick Disease Type B
NPC1	Niemann-Pick Disease Type C1
NPC2	Niemann-Pick Disease Type C2
PLS-DA	Partial Least Squares-Discriminant Analysis
SEM	Standard Error of the Mean
SRT	Substrate Reduction Therapy
TAG	Triacylglycerol
TLC	Thin Layer Chromatography
VIP	Variable Importance Projection
vLDL	Very-low-density lipoprotein.
