# Supplementary material for: Metabolomic Studies of Lipid Storage Disorders, with Special Reference to Niemann-Pick Type C Disease: A Critical Review with Future Perspectives
Source: Int J Mol Sci. 2020 Apr 5;21(7):2533. doi: 10.3390/ijms21072533 (PMC7178094; doi:10.3390/ijms21072533)
Supplement: Supplementary file 1 [file ijms-21-02533-s001.pdf]

Supplementary Materials: Metabolomic Studies of Lipid Storage Disorders, with Special Reference to Niemann-Pick Type C Disease: A Critical Review with Future Perspectives

Author List: Benita Claire Percival[1], Miles Gibson[1], Philippe B. Wilson[1], Frances M. Platt[2] and Martin Grootveld[1*]

Affiliations:

[1]Leicester School of Pharmacy, De Montfort University, The Gateway, Leicester, LE1 9BH

[2]Department of Pharmacology, University of Oxford, Mansfield Rd, Oxford, OX1 3QT, UK.

Author for Correspondence: Martin Grootveld

Email/Tel: mgrootveld@dmu.ac.uk/+44 (0)116 250 6443

ORCID: Benita Claire Percival (0000-0002-8769-8979); Philippe B. Wilson (0000-0003-0207-2246); Martin Grootveld (0000-0003-1502-3734); Frances Platt (0000-0001-7614-0403)

Ethical Approval, Sample preparation and NMR Acquisition Parameters

The investigation was carried out abiding by the rules of the Declaration of Helsinki 1975 (revised in 2013). All subjects gave their informed consent for inclusion before they participated in the study. De Montfort University, Leicester provided ethical approval for the protocol of collection of plasma from control participants (reference no. 1936). The GM1 Type II patients were consented under protocol 02-HG-0107 "Neurodegeneration in Glycosphingolipid Storage Disorders" under the National Human Genome Research Institute IRB.

Preparation for this set of samples involved centrifuging 500 μl volumes of plasma and removing 350 μl of the clear supernatant for analysis. A 50 μl aliquot of 1.00 M phosphate buffer (pH 7.00) also containing 0.05% (w/v) of the microbicide sodium azide was added to the supernatant, as was a 100 μl volume of ^2H_2O. This mixture was then rotamixed and added to 5-mm diameter NMR tubes. Samples were analysed using a Bruker AVII 700 MHz NMR spectrometer equipped with a ^1H TCI cryoprobe at an operating frequency of 699.989 MHz and a probe temperature of 298 K. Spectral acquisition involved the collection of 65,536 data points using 32 scans across a spectral width of 1,599 ppm. The CPMG pulse sequence was employed in order to suppress the broad protein signal envelope (which obscures the visibility and hence quantification of many low-molecular-mass metabolite resonances).
